# Organic Fluorescent Compounds that Display Efficient Aggregation-Induced Emission Enhancement and Intramolecular Charge Transfer

**DOI:** 10.3390/molecules23061446

**Published:** 2018-06-14

**Authors:** Ruibin Hou, Baohua Zhao, Yan Xia, Dongfeng Li

**Affiliations:** 1 School of Chemistry and Life Science, Changchun University of Technology, Changchun 130012, China; hrb1018@163.com (R.H.); zbh1969@126.com (B.Z.); 2 Advanced Institute of Materials Science, Changchun University of Technology, Changchun 130012, China

**Keywords:** 1,3,4-oxadiazole, aggregation-induced emission enhancement, blue light-emitting material

## Abstract

A series of symmetric sulfone-linked organic fluorescent compounds (**1a**–**c**) was synthesized and characterized. V-shaped **1a**–**c** were designed as aggregate of intramolecular charge transfer (ICT) and aggregation-induced emission enhancement (AIEE) processes. The **1a**–**c** emitted intense blue violet lights in normal solvents. A large red shift of the emission wavelength and dramatic decrease of emission efficiency occurred with increasing solvent polarity. The **1a**–**c** will function well as electron transport and blue light-emitting materials through theoretical calculations.

## 1. Introduction

Fluorescent organic molecules are widely used as active elements in organic light-emitting diodes (OLED), chemosensors, and bioprobes [1,2,3,4,5]. However, although most of these fluorescent organic molecules possess planar conjugated structures and display reasonable emission in dilute solutions, their emission is weak or nonexistent in the solid state because of aggregation-caused quenching. Such quenching limits the applications of fluorescent organic molecules [6,7,8]. Recently, multiple groups have independently reported an interesting phenomenon called aggregation-induced emission (AIE), wherein aggregated molecules emit more intense fluorescence than their dilute solutions [9,10,11]. Propeller-shaped molecules such as hexaphenylsilole, tetraphenylethene, and quinoline-malononitrile and their derivatives are known for their unique ability to promote AIE [12].

The integration of AIE and intramolecular charge transfer (ICT) into one molecule has attracted much attention. This type of luminophore can emit strong fluorescence not only in the aggregated form or solid state but also in dilute solution [13,14,15]. For example, Tang and coworkers developed a new donor (D)–acceptor (A) system that exhibits dual photoluminescence (PL) activity, including ICT and AIE [16]. However, the integration of AIE and ICT into one molecule is still rare and poorly understood, and thus warrants further study to clarify the relationship between molecular structure and optical properties.

Herein, we report a series of new V-shaped D-π-A-π-D fluorophores (**1a**–**c**) that exhibit strong blue emission in both solution (nonpolar solvents) and the solid state. We integrated a sulfone into **1a**–**c** because it has been demonstrated that sulfones possess a special V-shaped structure, which is helpful to weaken π–π stacking. A 1,3,4-oxadiazole was also included in **1a**–**c** because it is an attractive electron-accepting material that has received considerable interest for use in OLEDs.

## 2. Results and Discussion

### 2.1. Synthesis of Target Compounds ***1a***–***c***

**Scheme 1:**
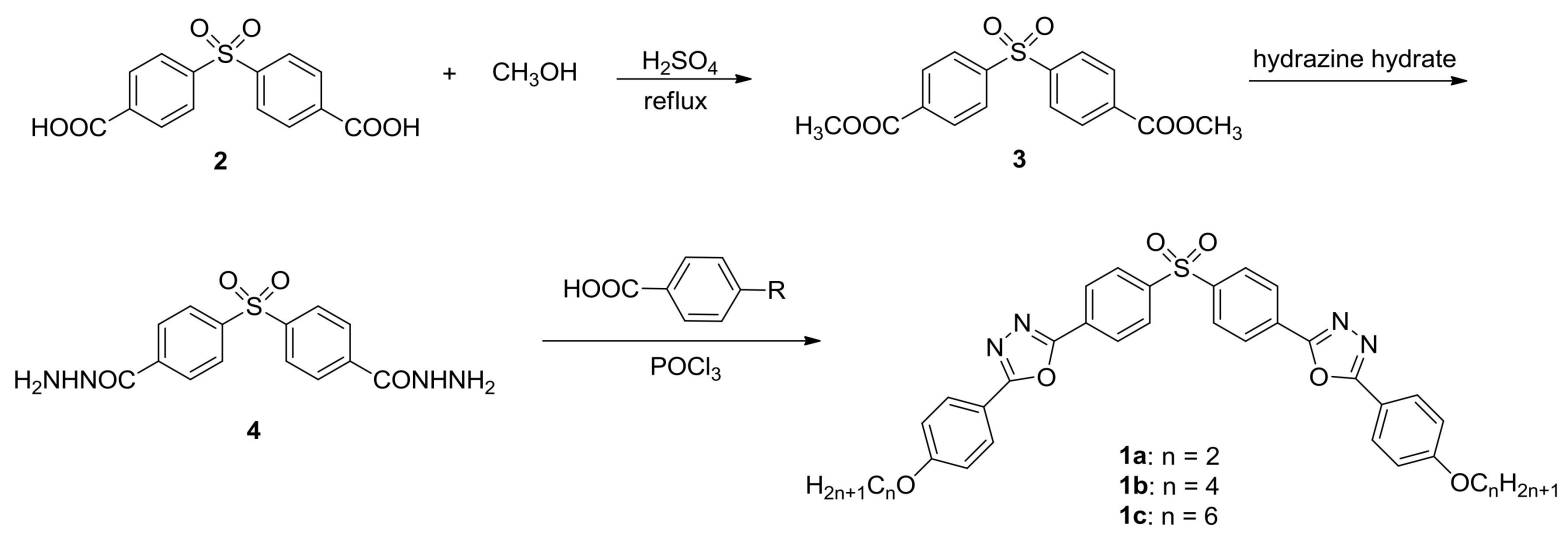
Synthetic route to **1a**–**c**.

The synthetic routes to **1a**–**c** are described in Scheme 1. The intermediate p,p’-dihydrazinocarbonyl diphenylsulfone (**4**) was synthesized according to a previous report [13]. The target compounds **1a**–**c** were obtained by condensation reactions between intermediate **4** and 4-alkoxybenzoic acid in phosphoryl chloride under reflux. Compounds **1a**–**c** were all white crystals and their yields ranged from 37% to 43%. The structures of **1a**–**c** were characterized by ^1^H and ^13^C-NMR spectroscopy, mass spectrometry, and elemental analysis.

### 2.2. The Optical Properties of Compounds ***1a***–***c***

**Figure 1:**
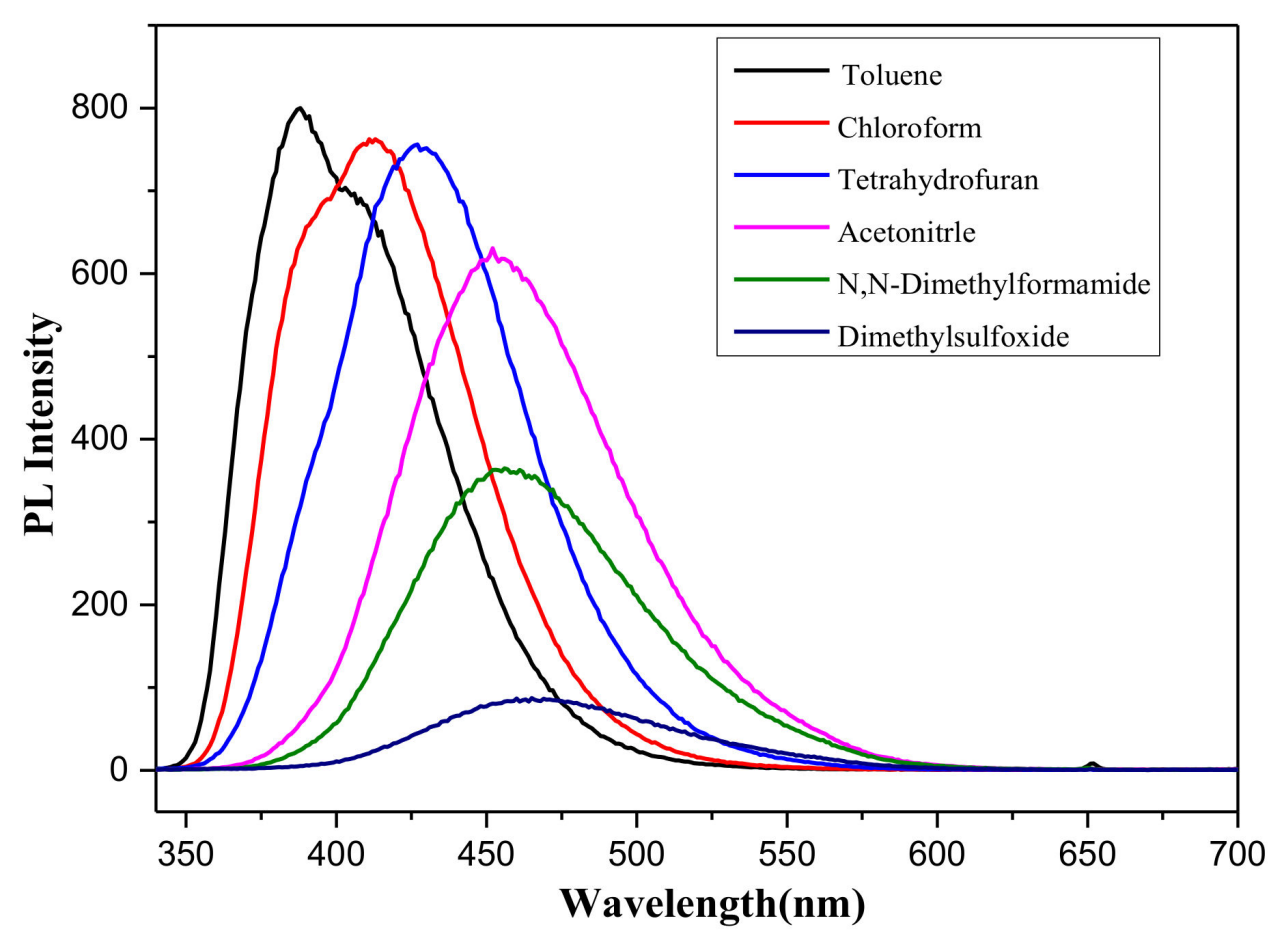
PL emission spectra of **1a** in different solutions at the same concentration (10^−5^ mol/L^−1^, excitation wavelength: 322 nm).

The optical properties of **1a**–**c** in solution and the solid state were investigated by UV-vis absorption and PL spectroscopies. The data revealed that the alkyl chain did not influence the properties of these compounds. Figure S5 presents the UV-vis absorption spectra of the same concentration of **1a**–**c** in different solvents. The absorption maxima (λ_ab_) exhibit a slight red shift of a few nanometers with increasing solvent polarity. In contrast, the PL maxima of **1a**–**c** vary dramatically with changes in solvent polarity, as shown in Figure 1 and Figure S6. The PL intensity gradually decreased with increasing solvent polarity and fluorescent quantum yields (Q_ys_) decreased from 0.42 to 0.05 when the solvent was changed from toluene to DMSO. Emission maxima (λ_em_) red-shifted from 387 nm in toluene to 468 nm in DMSO. The red shift and decreased QY can be ascribed to stabilization of the excited state. This behavior originates from the strong ICT character from the alkoxyl benzene to the sulfone. This result indicates that the ICT excited state has a larger dipole moment than the ground state, which is caused by substantial charge redistribution. Because of the dipole–dipole interaction of a polar solvent and excited state, the ICT state is more stable in a polar solvent than in a nonpolar one.

**Figure 2:**
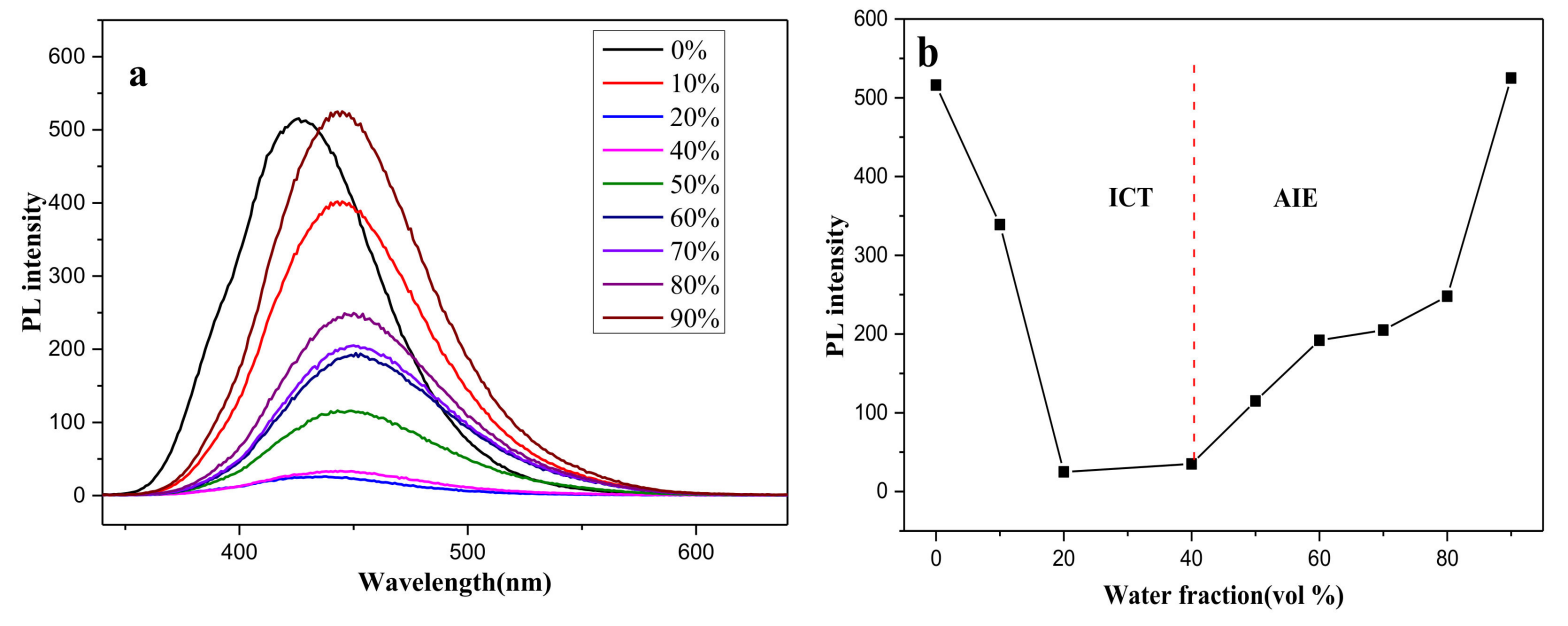
(**a**) PL spectra of **1a** in THF/water mixtures with different water fractions (ƒ_w_); and (**b**) plot of peak intensity of **1a** versus ƒ_w_ in the THF/water mixtures (10^−5^ mol·L^−1^, excitation wavelength: 322 nm).

To determine whether **1a**–**c** are AIE-active, fluorescence emission spectra of their diluted mixtures in THF/water and DMF/water mixtures with different water fractions (ƒ_w_) were measured (Figure 2 and Figure S7). Figure 2 reveals that **1a** in pure THF solution emitted intense indigo blue fluorescence at 427 nm, which was dramatically quenched when a small amount of water was added, as evidenced by its weak emission in the THF/water mixtures with ƒ_w_ ≤ 40 vol%. Meanwhile, λ_em_ red-shifted to about 449 nm upon addition of water, which was mainly attributed to the increase of solvent polarity and enhancement of the ICT effect. If a hydrophobic environment was created inside the aggregates, the ICT effect should be alleviated, and the fluorescence should recover because of the AIE effect. The pure DMF solution of **1a** displayed weak blue fluorescence at λ_em_ = 458 nm. When ƒ_w_ of the DMF/water mixture was low, λ_em_ of **1a** was dramatically quenched and slightly blue-shifted. When ƒ_w_ of the DMF/water mixture was high, λ_em_ of **1a** was red-shifted and the fluorescence intensity did not recover. In this case, the solvent polarity influences the AIE effect. These data illustrate our speculation that **1a** is a luminogen with both ICT and AIE characteristics. Both **1b** and **1c** exhibited similar AIE effects, as shown in Figure S7.

**Figure 3:**
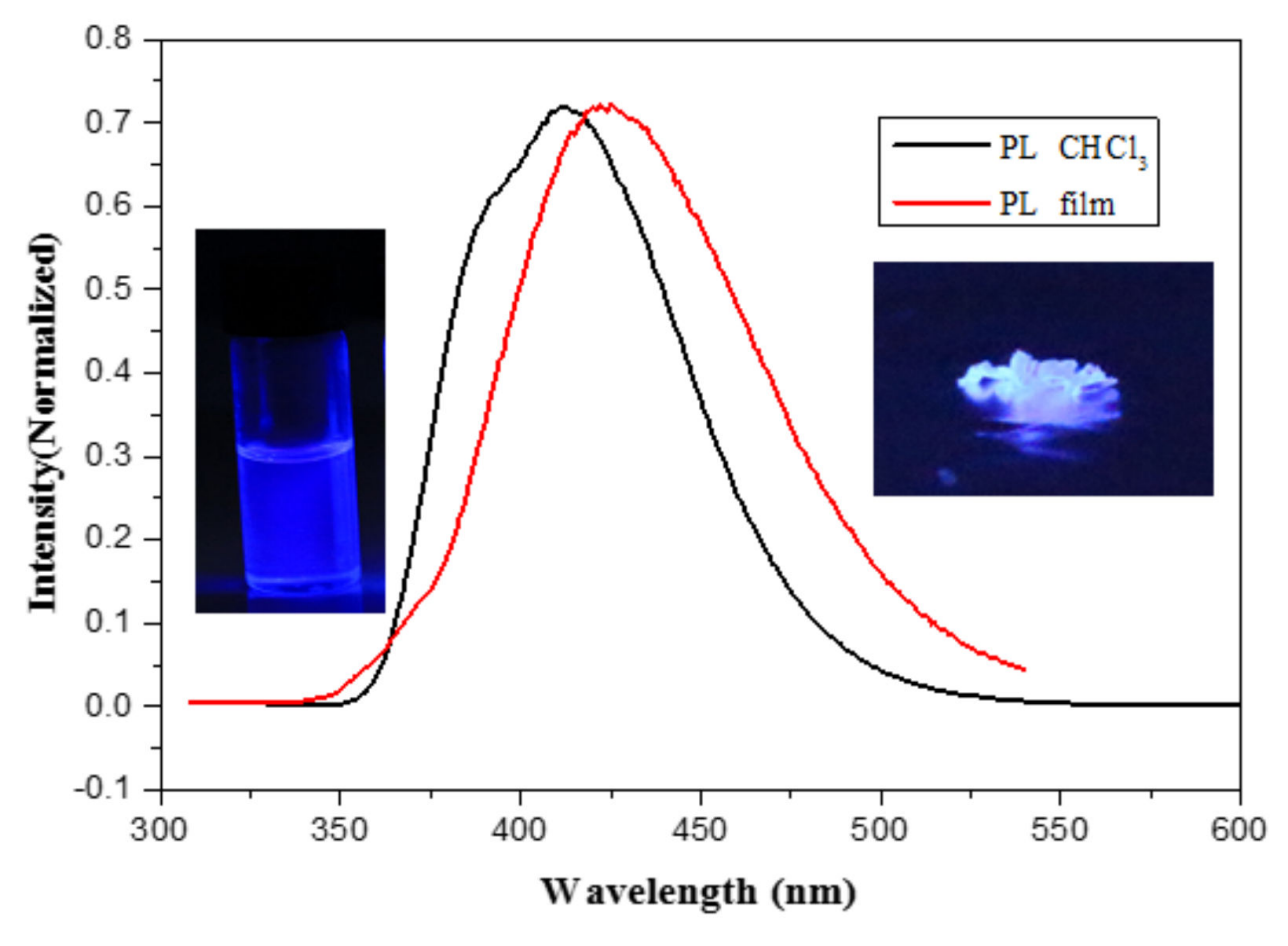
PL spectra of **1a**. Inset are photographs of ground powder of **1a** and **1a** in toluene under 365 nm illumination.

Such AIE properties were also confirmed by the vivid image of the solid powders under 365 nm UV illumination, as depicted in Figure 3. The solid powder and **1a** dissolved in toluene emitted strong blue fluorescence. Conversely, the emission spectra of the solution and film samples were different, considering the broad emission peaks and red shift in Figure 3. Compound **1a** exhibited dual-state emission behaviors and that demonstrated its fluorescence was strong in both solution and the aggregated state.

### 2.3. The Electrochemical Properties of ***1a***–***c***

**Figure 4:**
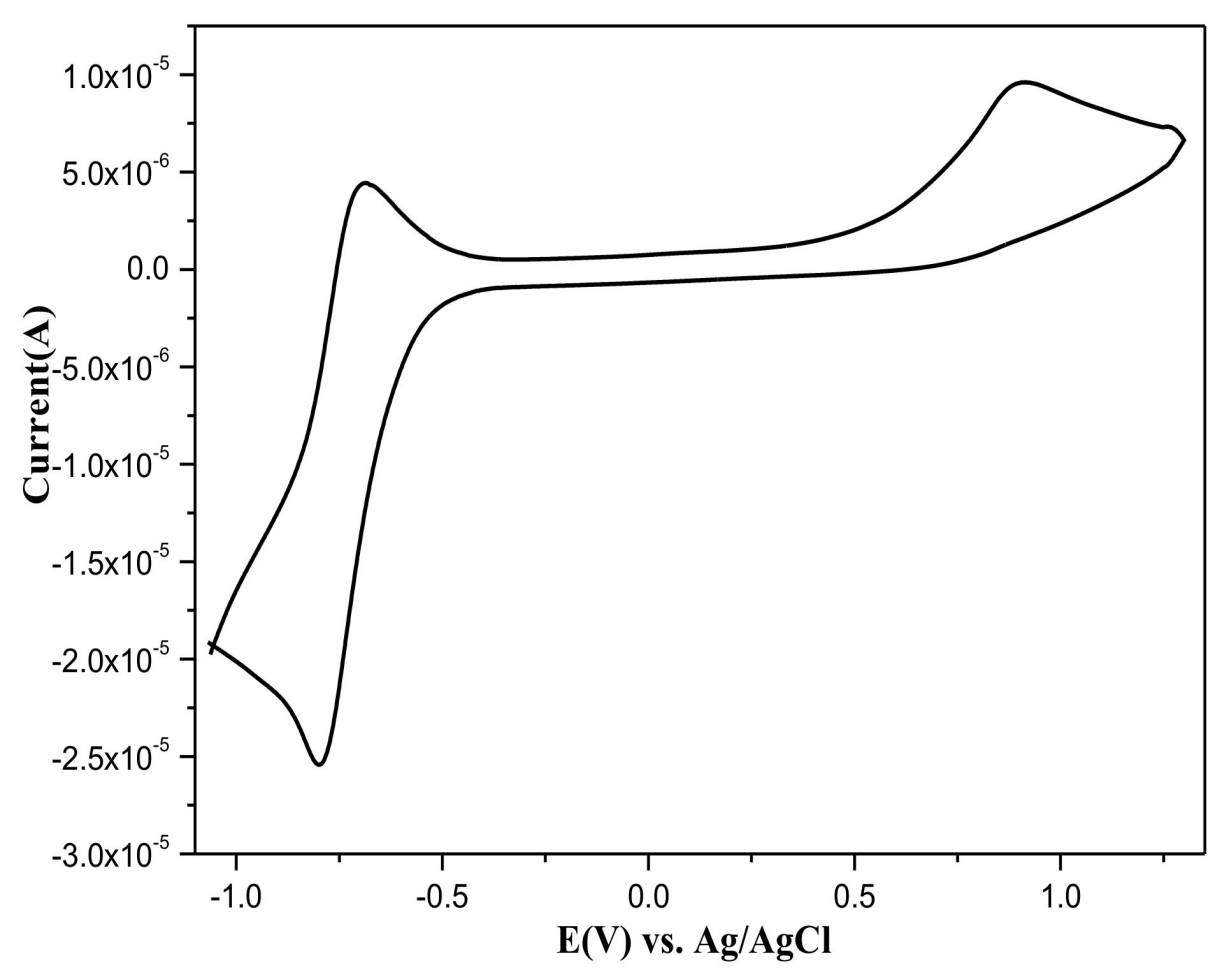
Cyclic voltammograms of **1a** in CHCl_3_ (1 × 10^−3^ M).

The electrochemical properties of **1a**–**c** in CHCl_3_ were investigated by CV. The electrochemical data are summarized in Table 1. Figure 4 displays representative CV traces for the oxidation and reduction of **1a**, demonstrating that **1a** undergoes a quasi-reversible reduction at the cathodic potential associated with the reduction of the electron-deficient sulfone moiety to form an anion radical. Compound **1a** also exhibits an irreversible oxidation process corresponding to the removal of electrons from the terminal alkoxy phenyl group to form a radical cation.

### 2.4. Theoretical Calculations

All calculations were carried out using the Gaussian 09 package [18]. Calculations of the electronic ground state of compound **1a**–**c** were carried out using density functional theory (DFT) [19] with the B3LYP exchange-correlation functional [20,21] together with the 6–31 G basis set [22]. GaussView 5.0.8 was used to calculate the interface of the structures and manipulate orbital.

**Figure 5:**
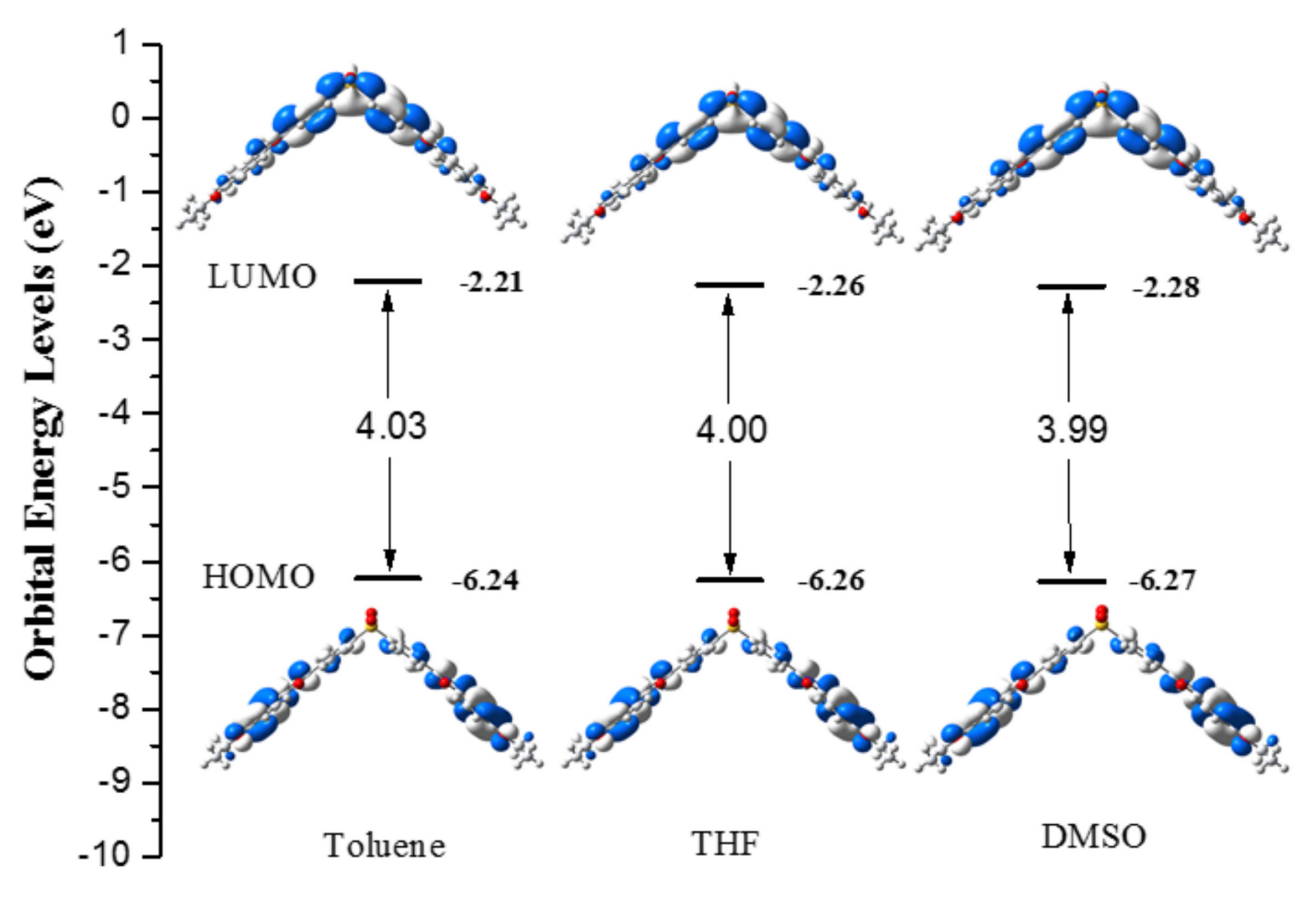
Electron density contours and orbital energies calculated for the HOMO and LUMO of **1a** at the Beck3LYP/DZP level in toluene, THF and DMSO.

DFT was used to calculate molecular orbitals to understand the photophysical behavior of the synthesized compounds. To estimate the charge-carrier injection barriers, we investigated the changes that occurred upon frontier orbital substitution. Figure 5 shows the highest occupied molecular orbital (HOMO), lowest unoccupied molecular orbital (LUMO) and energy gap (*E*_g_) of **1a** in toluene, THF and DMSO. The electron density distributions of representative **1a** change considerably between the HOMO and LUMO. The electron density distributions of the HOMO are mostly located over the alkoxy benzene groups, whereas the LUMOs are mainly concentrated on the sulfone core. *E*_g_ between the HOMO and LUMO decreases from 4.03 to 3.97 eV from toluene to DMSO, which is consistent with the slight red shift of 1 nm of the UV-vis absorption peak in polar solvents. Generally, this type of dye molecule with such an electron distribution indicates ICT character, which is consistent with the experimental results.

The calculated vertical ionization potential IP (IP_v_), adiabatic IP (IP_a_), vertical electron affinity (EA_v_), and adiabatic electron affinity (EA_a_) for **1a** are listed in Table 2. The reorganization energy (λ) can be used to estimate the charge transport rate and balance between holes and electrons; these results are also provided in Table 2. The reorganization energy for electron transport can be expressed in terms of the electron extraction potential (EEP): λ_e_ = EEP − EA_v_. EEP represents the electron extraction potential, which is the energy difference between M and MM- (anionic) when considering M-. It was found that representative **1a** had a low λ_e_ value of 0.28, which is similar to that of tris(8-hydroxyquinolinato)aluminum (λ_e_ = 0.276) [23] and good electron transfer ability. These results indicate that **1a** may be a promising electron transport material.

The thermal properties of **1a**–**c** were evaluated by thermal gravimetric analysis (TGA) and differential scanning calorimetry (DSC) under nitrogen atmosphere. (Table 1 and Figure S4). The TGA revealed that the compounds possessed relatively high thermal stability with an initial weight loss (5%) temperature of 383–396 °C. The glass transition temperatures of **1a**–**c**, as shown in Table 1, were 119 °C, 156 °C and 145 °C, respectively. The excellent thermal stability of **1a**–**c** should aid the formation of amorphous films.

## 3. Experimental

### General Information

Ultraviolet-visible (UV-vis) spectra were recorded on a Lambda 25 spectrophotometer. Fluorescence spectra were obtained using a Shimadzu RF-5301PC fluorescence spectrophotometer. Cyclic voltammetric studies were carried out using a CHI 852C instrument with CHCl_3_ as the solvent (10^−3^ M) and 0.1 M Bu_4_NClO_4_ as the supporting electrolyte. Counter and working electrodes consisted of a Pt wire and glass carbon, respectively, and the reference electrode was Ag/AgCl. The thermal stability of the target compounds was characterized using a Shimadzu DTG-60H thermogravimetric analyzer.

Starting compound **2** and p,p’-Dihydrazinocarbonyl diphenylsulfone (**3**) were synthesized according to literature method [13].

Representative procedure for the synthesis of **1a**–**c**: A mixture of compound **2** (2.2 mmol), p,p’-Dihydrazinocarbonyl diphenylsulfone (1 mmol), and phosphorous oxychloride (8 mL) was heated at reflux for 8 h, and then poured onto crushed ice after cooling to room temperature. The white solid was obtained. The crude product was separated on a flash silica gel column (CHCl_2_/Ethyl acetate) to afford **1a**–**c**.

*p,p’-Bis[2-(4-Ethoxyphenyl)-1,3,4-oxadiazol-5-yl]diphenylsulfone* (**1a**): White powder, yield: 40%. Mp 329 °C (DSC). ^1^H-NMR (400 MHz, CDCl_3_): *δ* (ppm) 1.44 (t, *J* = 7.2 Hz, 6H), 4.11 (q, *J* =7.2 Hz, 4H), 7.01 (d, *J* = 8.8 Hz, 4H), 8.04 (d, *J* = 9.0 Hz, 4H), 8.12 (d, *J* = 9.0 Hz, 4H), 8.27 (d, *J* = 8.8 Hz, 4H); ^13^C-NMR (100 MHz, CDCl_3_): *δ* (ppm) 14.65, 63.85, 115.12, 115.61, 127.67, 128.60, 128.79, 128.94, 143.37, 162.21, 162.51, 165.45; FTIR (KBr, cm^−1^): 3079, 2920, 1615, 1489, 1066, 842; TOF-Ms (*m*/*z*) [M + H]^+^ = 596.1648; Anal. Calcd for C_32_H_26_N_4_O_6_S: C, 64.64. H, 4.41. Found: C, 64.71. H, 4.48.

*p,p’-Bis[2-(4-Butoxyphenyl)-1,3,4-oxadiazol-5-yl]diphenylsulfone* (**1b**): White powder, yield: 43%. Mp 325 °C (DSC). ^1^H-NMR (400 MHz, CDCl_3_): *δ* (ppm) 0.98 (t, *J* = 7.2 Hz, 6H). 1.52 (m, 4H), 1.79 (m, 4H), 4.03 (t, *J* = 6.4 Hz 4H), 7.01 (d, *J* = 8.8 Hz, 4H), 8.04 (d, *J* = 8.8 Hz, 4H), 8.13 (d, *J* = 8.8 Hz, 4H), 8.28 (d, *J* = 8.8 Hz, 4H); ^13^C-NMR (100 MHz, CDCl_3_): *δ* (ppm) 13.79, 19.19, 31.14, 68.06, 115.14, 115.54, 127.67, 128.60, 128.80, 128.92, 143.37, 162.43, 162.50, 165.47; FTIR (KBr, cm^−1^): 3080, 2920, 1620, 1486, 1069, 840; TOF-Ms (*m*/*z*) [M + H]^+^ = 651.1227; Anal. Calcd for C_36_H_34_N_4_O_6_S: C, 66.45. H, 5.27. Found: C, 66.46. H, 5.34.

*p,p’-Bis[2-(4-Hexoxyphenyl)-1,3,4-oxadiazol-5-yl]diphenylsulfone* (**1c**): White powder, yield: 22%. Mp 282 °C (DSC). ^1^H-NMR (400 MHz, CDCl_3_): *δ* (ppm) 0.91 (t, *J* = 7.2 Hz, 6H), 1.38 (m, 24H), 1.83 (m, 4H), 4.05 (t, *J* = 6.4 Hz, 4H), 7.03 (d, *J* = 8.8 Hz, 4H), 8.06 (d, *J* = 8.8 Hz, 4H), 8.14 (d, *J* = 8.8 Hz, 4H), 8.29 (d, *J* = 8.8 Hz, 4H); ^13^C-NMR (100 MHz, CDCl_3_): *δ* (ppm) 14.04, 22.63, 25.72, 29.12, 31.59, 68.44, 115.20, 115.59, 127.73, 128.67, 128.87, 128.98, 143.42, 162.49, 165.54; FTIR (KBr, cm^−1^): 3080, 2924, 1622, 1486, 1069, 844; TOF-Ms (*m*/*z*) [M + H]^+^ = 707.1256; Anal. Calcd for C_40_H_42_N_4_O_6_S: C, 67.97, H, 5.99. Found: C, 67.99. H, 6.03.

## 4. Conclusions

A series of D-π-A-π-D type sulfone derivatives with alkoxy D groups and sulfone A units was produced. The experimental results demonstrate that these V-shaped compounds display the features of both ICT and AIE. These blue emitters are solution processible and exhibit good film-forming ability and high thermal stability, marking them promising as electron transport materials.

## Figures and Tables

**Table 1:** Photophysical, physical, and electrochemical data for **1a**–**c**.

Comp.	^a^ λ_max,Abs_ (nm)	^a^ λ_max,em_ (nm)	^b^ λ_max,em_ (nm)	^c^ *Φ*_F_	E^red/peak^ (V)	E_1_^ox/peak^ (V)	^d^*E*_g_^opt^ (eV)	^e^ T_g_/T_5d_ (°C)
**1a**	323	412	420	0.42	−0.73	0.62	4.18	119/396
**1b**	326	411	423	0.36	−0.74	0.75	4.21	156/383
**1c**	328	411	423	0.32	−0.76	0.78	4.26	145/386

^a^ Measured in CHCl_3_. ^b^ Measured for film. ^c^ Measured in CHCl_3_ with quinine sulfate as a standard (QY = 0.54) [17]. ^d^ Calculated using *E*g = 1240/λ^abs^onset. ^e^ Analyzed by DSC/TGA at a heating rate of 10 °C·min^−1^ under N_2_.

**Table 2:** Ionization potentials, electron affinities, extraction potentials, internal reorganization energies, and Δ = ⎹λ_h_ − λ_e_⎸ (in eV) for the studied complexes.

Comp.	IP (v)	IP (a)	HEP	EA (v)	EA (a)	EEP	λ_hole_	λ_electron_	Δ
**1a**	6.21	6.07	5.90	2.35	2.49	2.63	0.31	0.28	0.03
**1b**	6.18	6.07	5.90	2.29	2.49	2.63	0.28	0.34	0.06
**1c**	6.17	6.07	5.90	2.29	2.49	2.63	0.27	0.34	0.07
**Alq_3_**	IP (5.8)			EA (3.0)			0.242	0.276	0.034

## Supplementary Materials

Figures S1–S4: Characterization of compounds **1a**–**c**, Figure S5: Absorption spectra of **1a**–**c**, Figure S6: PL emission spectra of **1b**, Figure S7: PL emission spectra of **1a**,**1b** in DMF/H_2_O mixtures, Figure S8: Cyclic voltammograms of **1a**, Figure S9: Electron density contours and orbital energies calculated for the HOMOs and LUMOs of **1b**,**1c**.
