# Supplementary material for: Organic Fluorescent Compounds that Display Efficient Aggregation-Induced Emission Enhancement and Intramolecular Charge Transfer
Source: Molecules. 2018 Jun 14;23(6):1446. doi: 10.3390/molecules23061446 (PMC6100197; doi:10.3390/molecules23061446)
Supplement: Supplementary file 1 [file molecules-23-01446-s001.pdf]

Organic fluorescent compounds that display efficient aggregation-induced emission enhancement and intramolecular charge transfer

Ruibin Hou[1,2], Baohua Zhao[1], Yan Xia [1,2,*] and Dongfeng Li[1,*]

[1]*School of Chemistry and Life Science, Changchun University of Technology, Changchun, 130012, China*

[2]*Advanced Institute of Materials Science, Changchun University of Technology, Changchun 130012, China*

Figure S1-S4: Characterization of compounds **1a-c**.

Figure S5: Absorption spectra of **1a-c**.

Figure S6: PL emission spectra of **1b**.

Figure S7: PL emission spectra of **1a-b** in DMF/H_2O mixtures.

Figure S8: Cyclic voltammograms of **1a**.

Figure S9: Electron density contours and orbital energies calculated for the HOMOs and LUMOs of **1b-c**

Characterization

^{1}H NMR

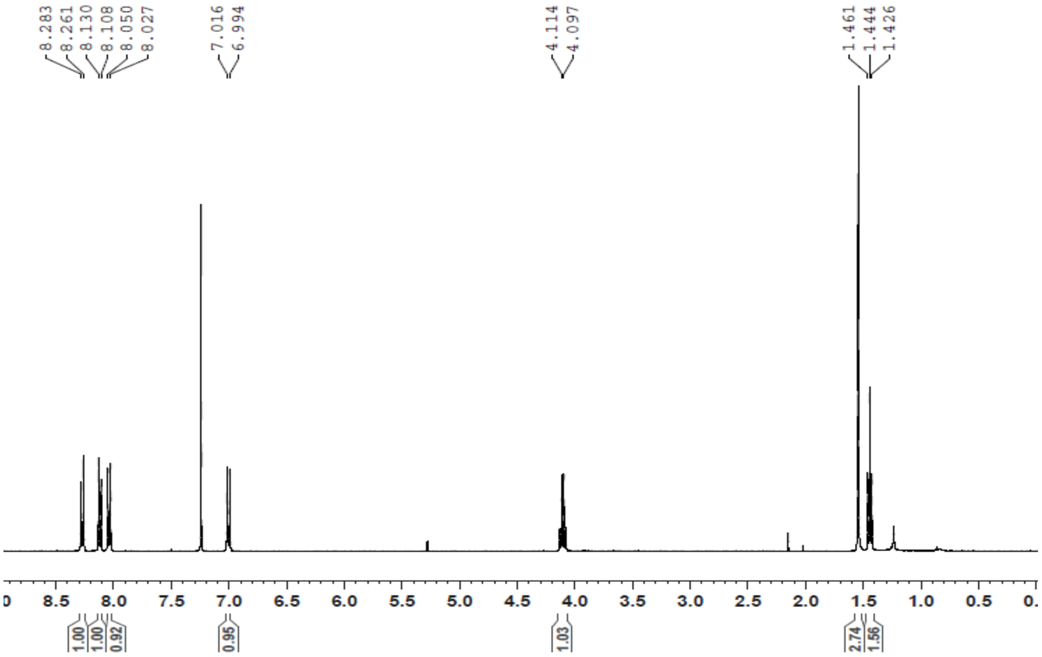

Figure S1 (1) ^{1}H NMR spectra of **1a**.

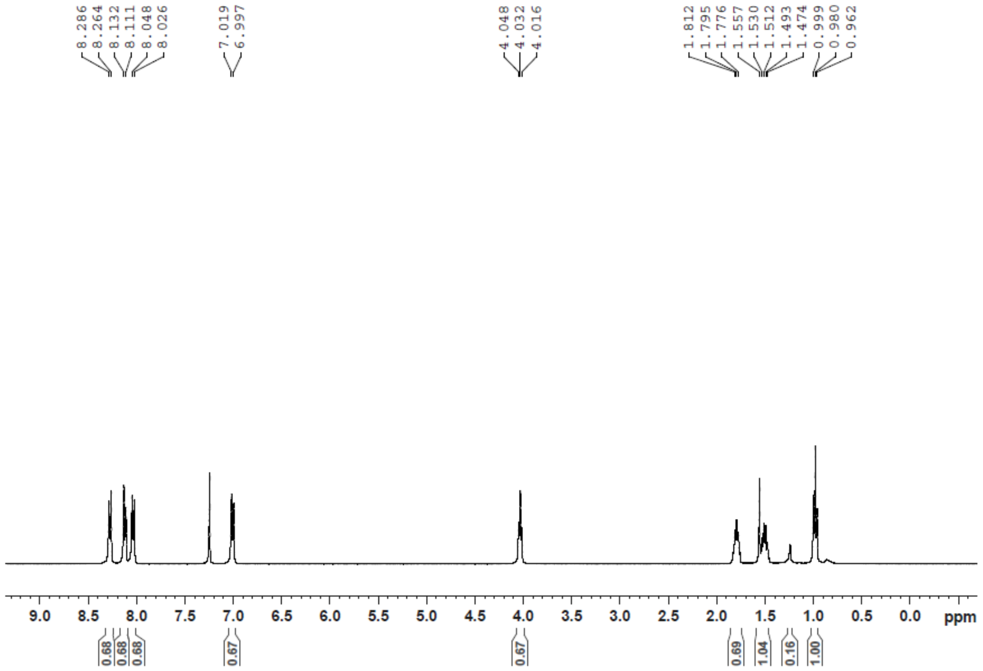

Figure S1 (2) ^{1}H NMR spectra of **1b**.

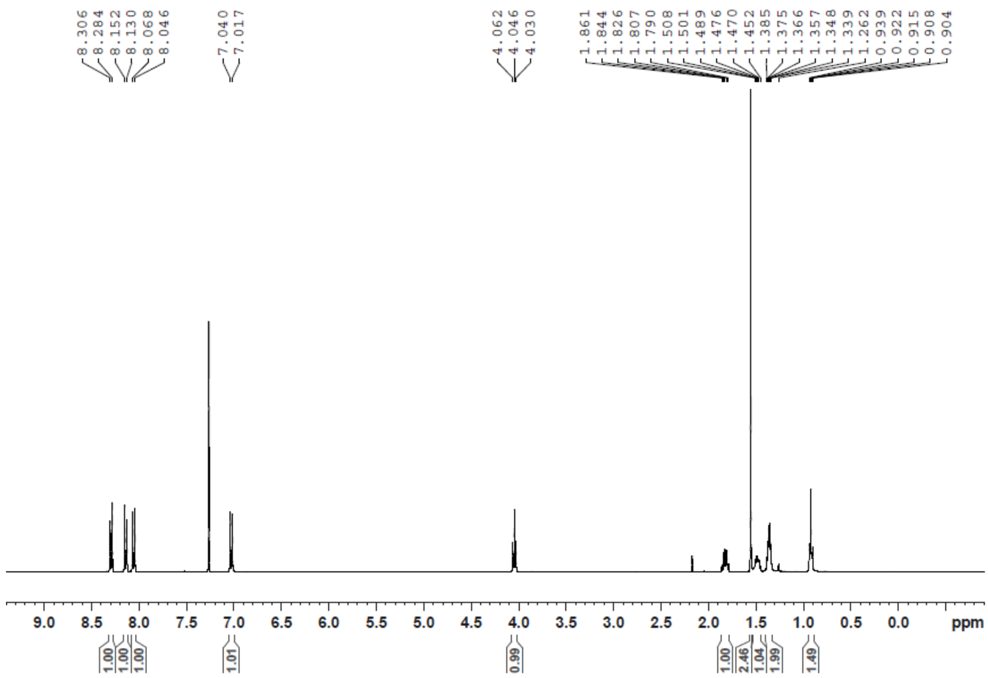

Figure S1 (3) ^{1}H NMR spectra of **1c**.

^{13}C NMR

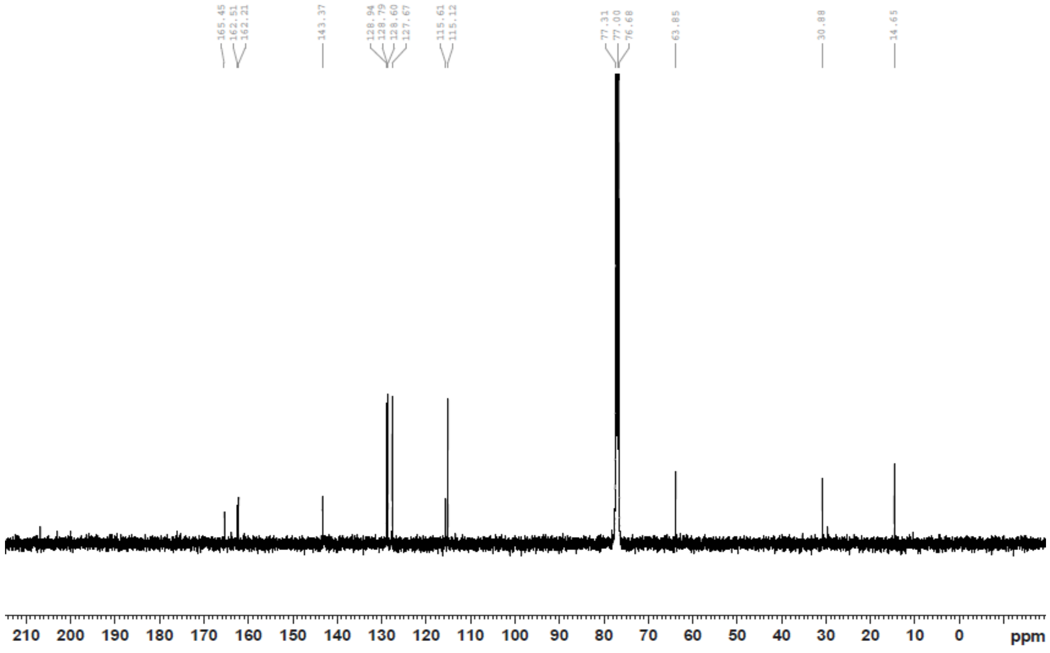

Figure S2 (1) ^{13}C NMR spectra of **1a**.

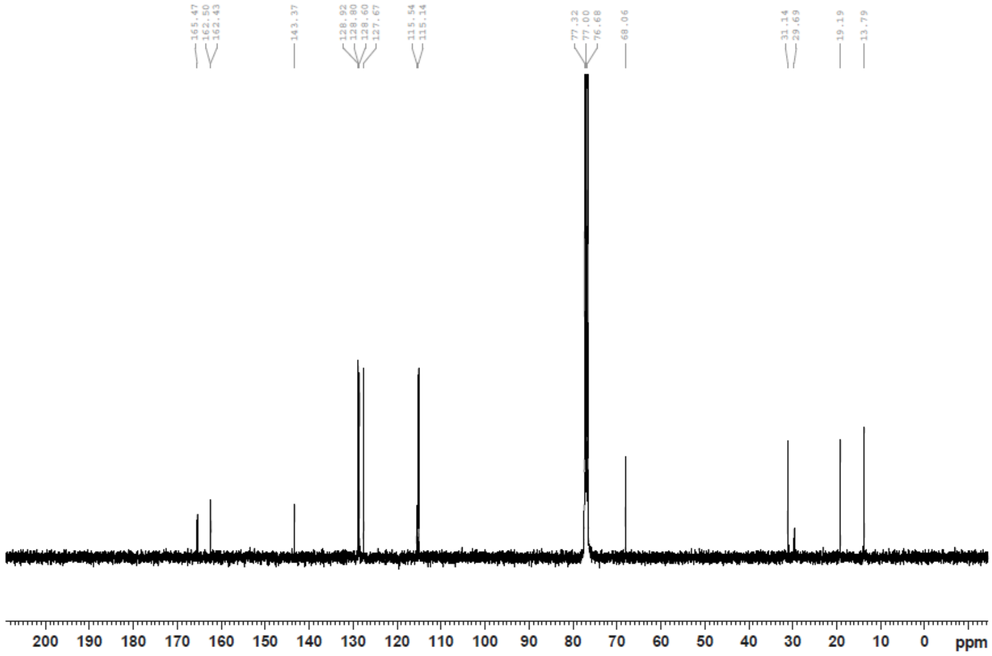

Figure S2 (2) ^{13}C NMR spectra of **1b**.

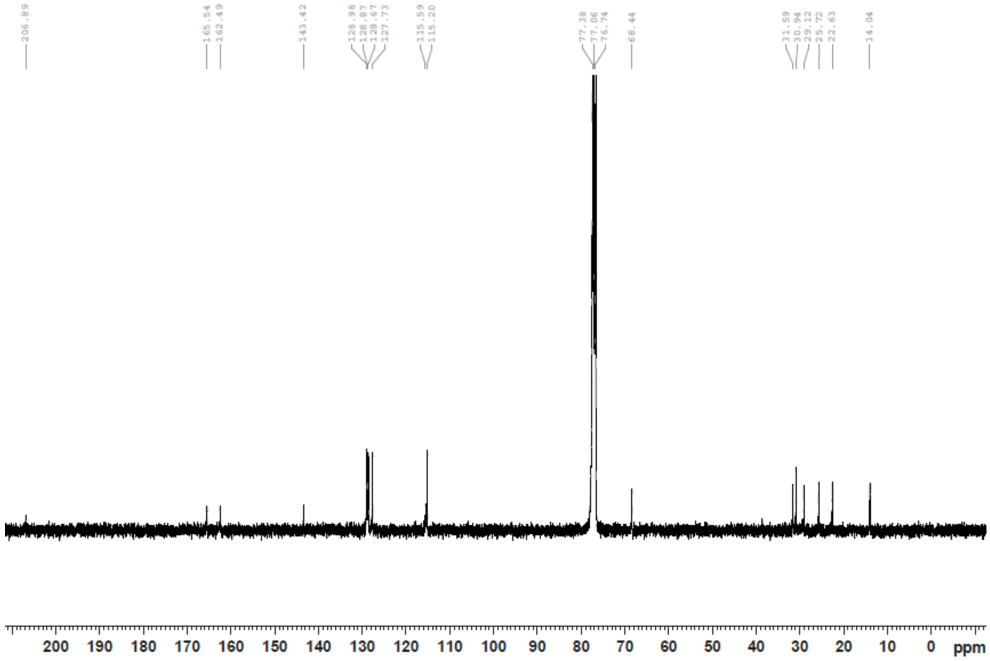

Figure S2 (3) ^{13}C NMR spectra of **1c**.

FT-IR

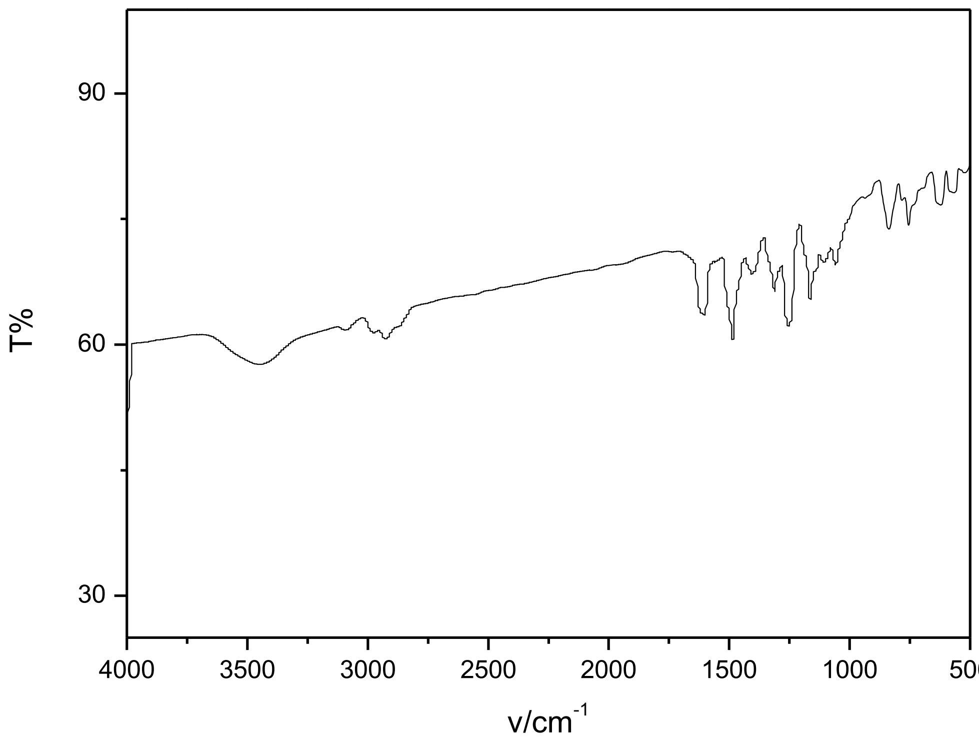

Figure S3 (1) IR spectra of **1a**.

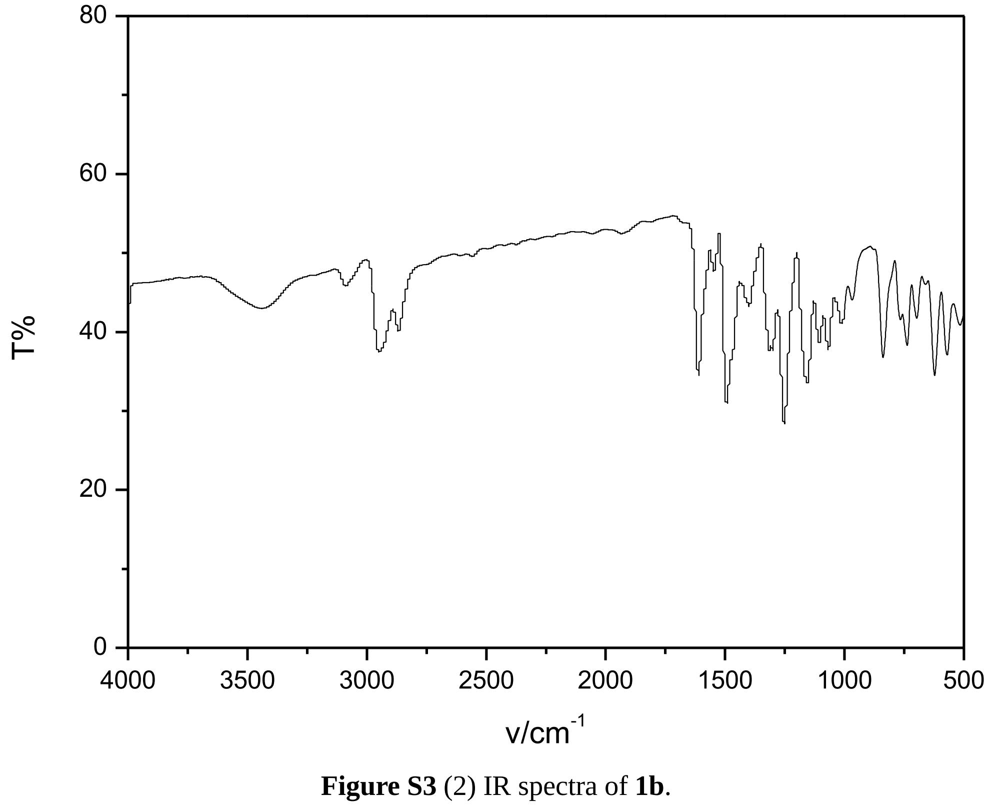

Figure S3 (2) IR spectra of **1b**.

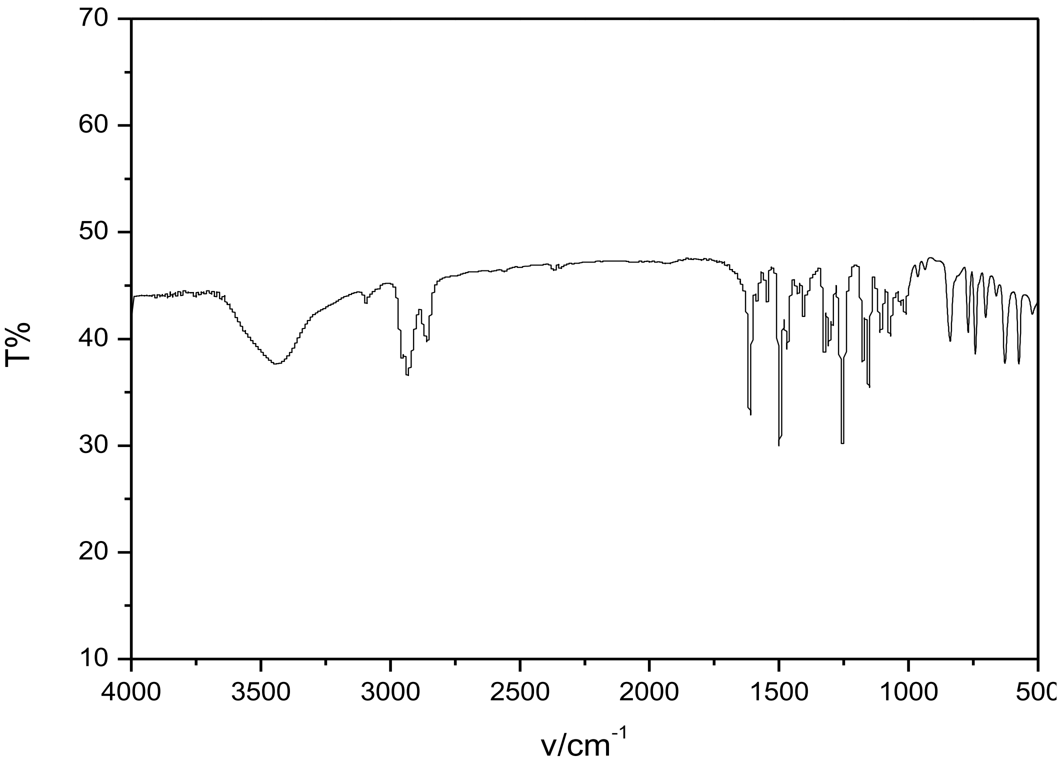

Figure S3 (3) IR spectra of **1c**.

TOF Ms

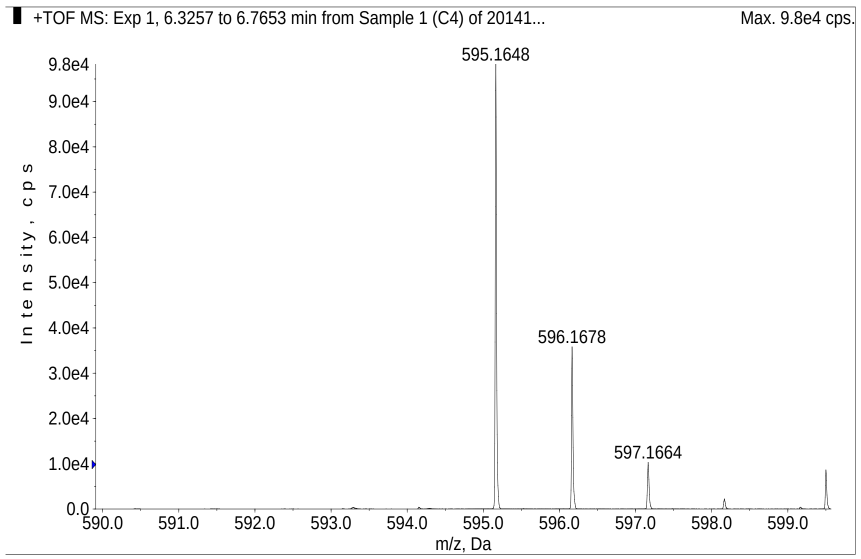

Figure S4 (1) TOF MS spectra of **1a**.

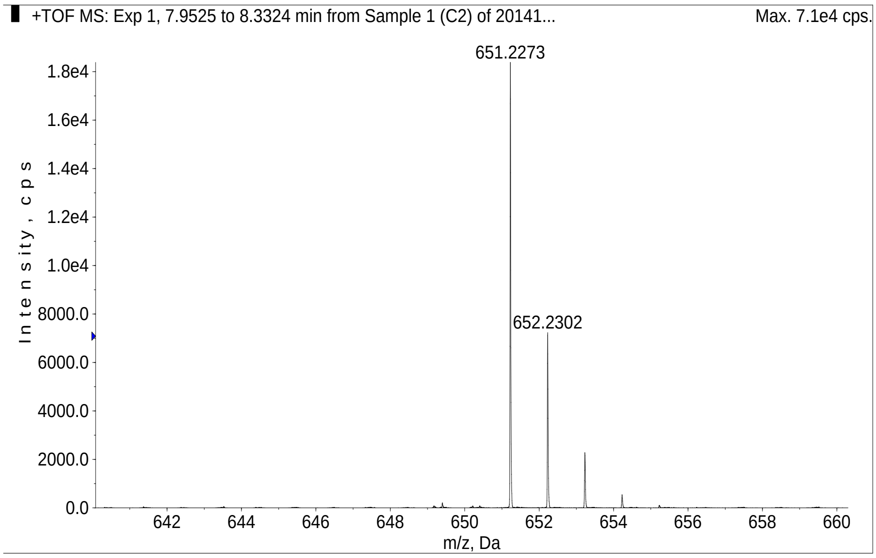

Figure S4 (2) TOF MS spectra of **1b**.

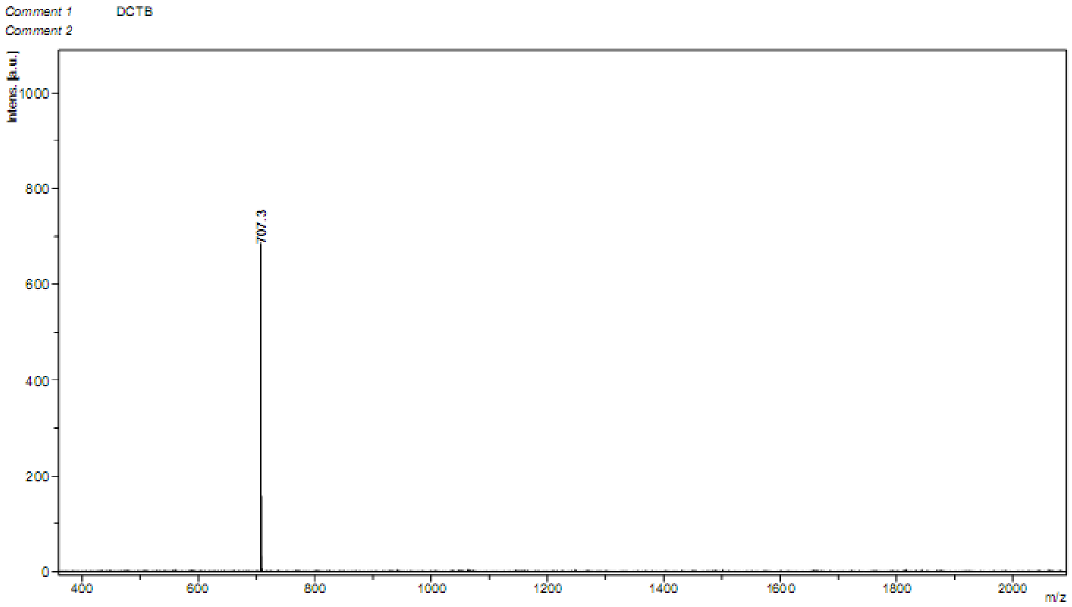

Figure S4 (3) TOF MS spectra of **1c**.

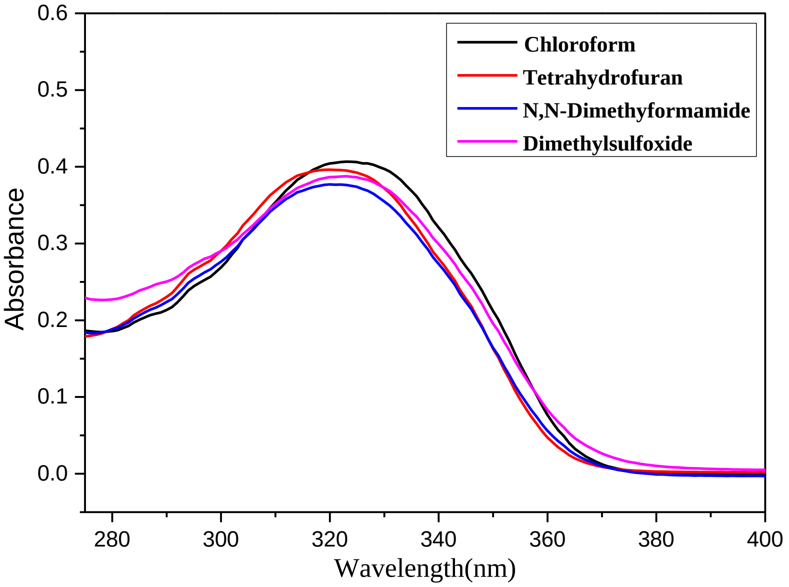

Figure S5 (1) Absorption spectra of **1a** in different solutions at the same concentration(10^{-5}molL^{-1}).

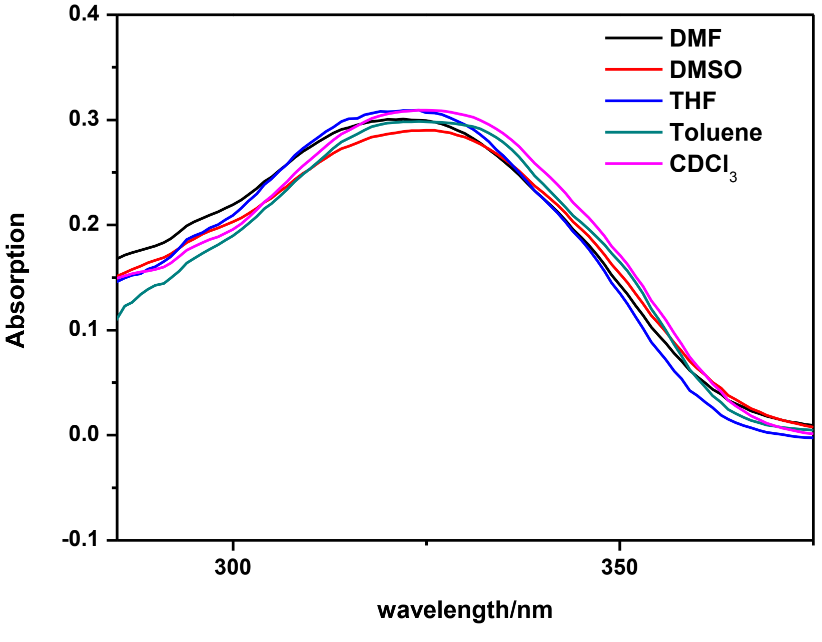

Figure S5 (2) Absorption spectra of **1b** in different solutions at the same concentration(10^{-5}molL^{-1}). DMSO=dimethylsulfoxide.

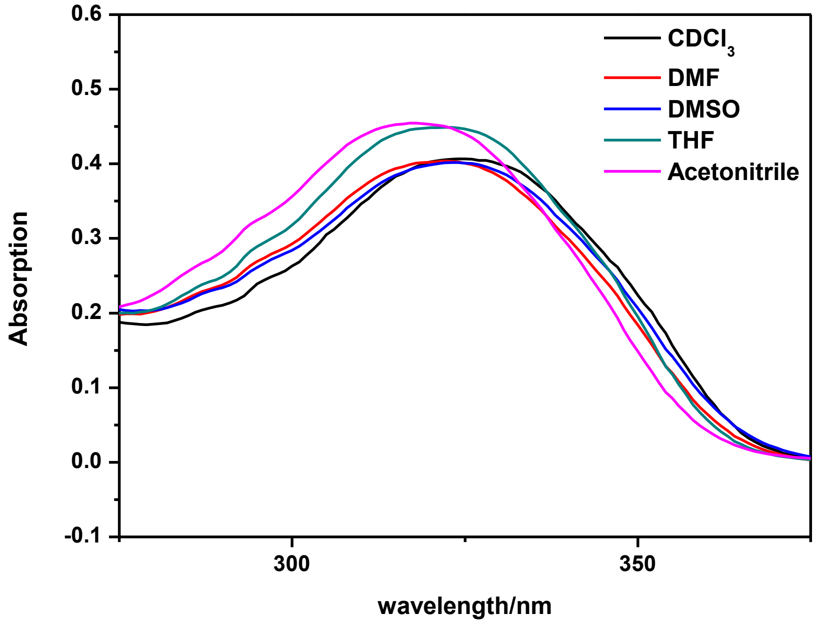

Figure S5 (4) Absorption spectra of **1c** in different solutions at the same concentration(10^{-5}molL^{-1}). DMSO=dimethylsulfoxide.

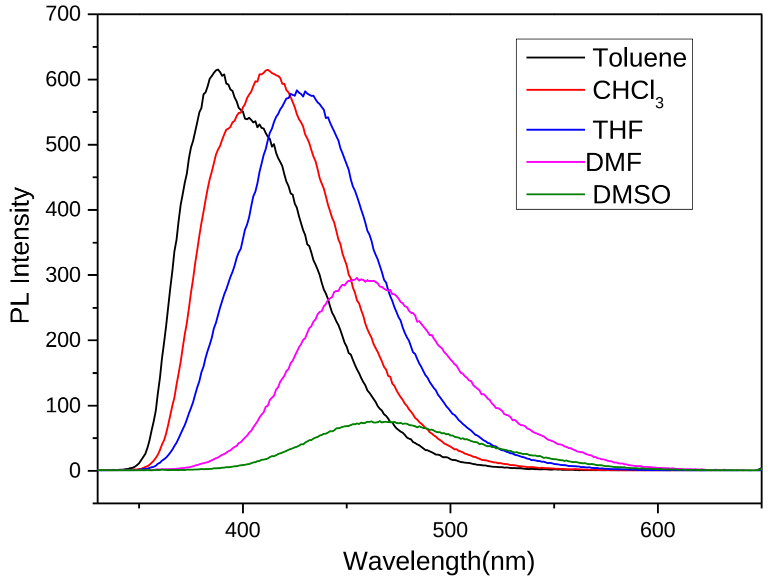

Figure S6 (1) PL emission spectra of **1b** in different solutions at the same concentration (10^{-6}molL^{-1}, excitation wavelength:327nm). DMSO=dimethylsulfoxide.

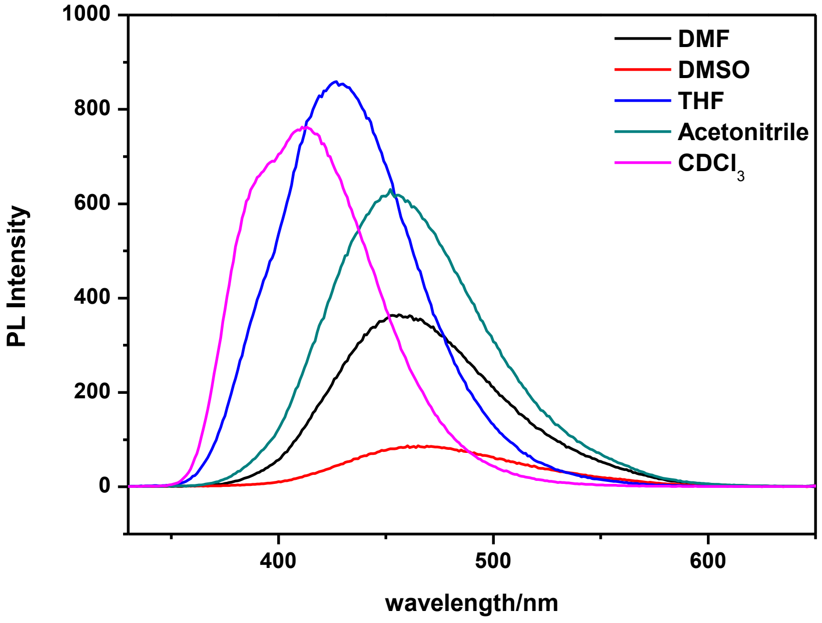

Figure S6 (1) PL emission spectra of **1b** in different solutions at the same concentration (10^{-5}molL^{-1}, excitation wavelength:325nm). DMSO=dimethylsulfoxide.

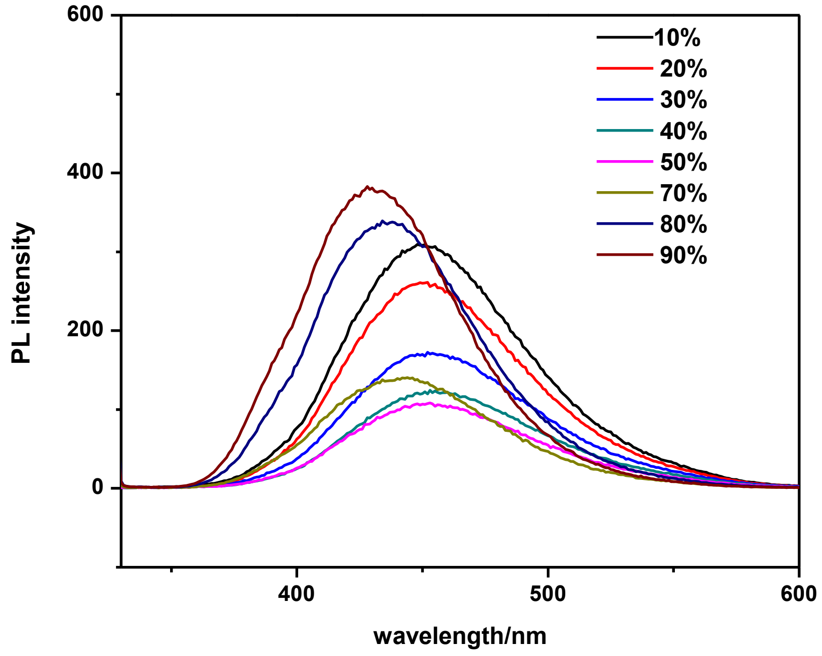

Figure S7 (1) PL emission spectra of **1b** THF/H_2O mixtures at the same concentration (5×10^{-6}molL^{-1}, excitation wavelength:325nm).

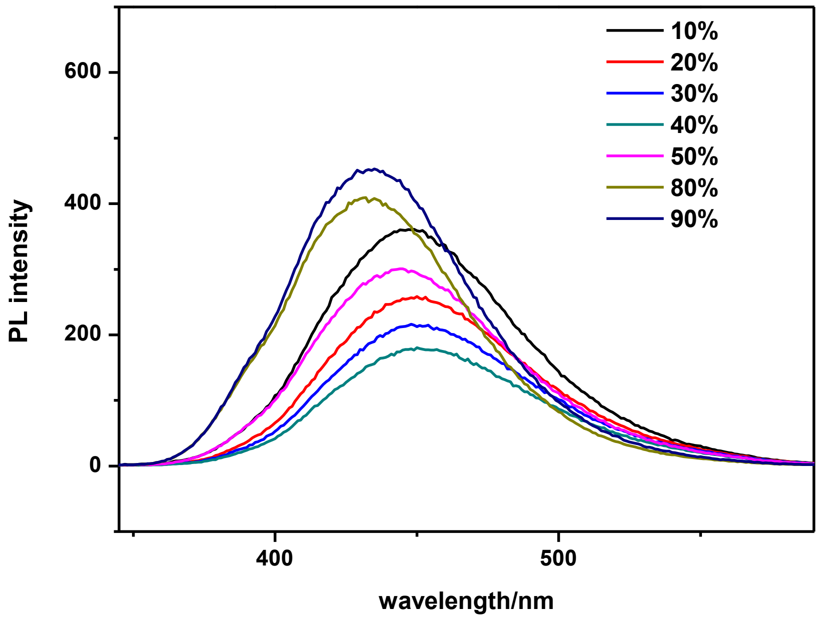

Figure S7 (2) PL emission spectra of **1b** THF/H_2O mixtures at the same concentration (10^{-6}molL^{-1}, excitation wavelength:327nm).

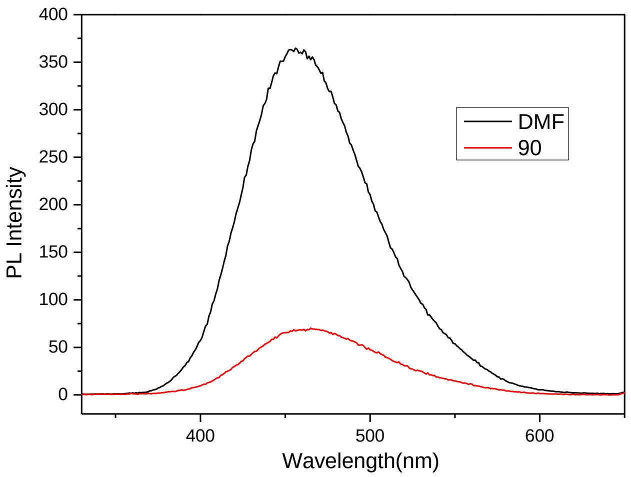

Figure S7 (3) PL emission spectra of **1a** DMF/H_2O mixtures at the same concentration (10^{-5}molL^{-1}, excitation wavelength:325nm).

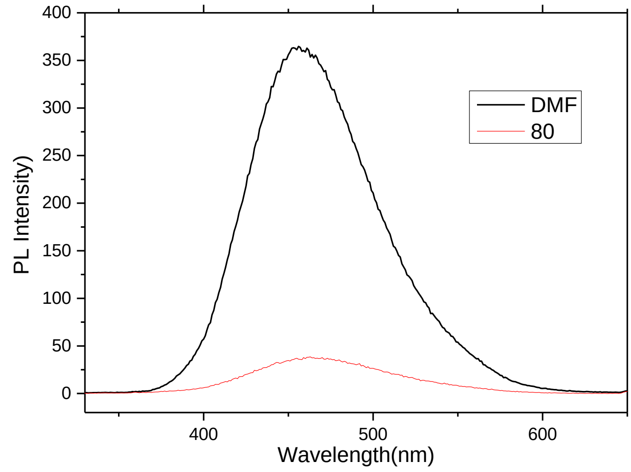

Figure S7 (4) PL emission spectra of **1b** DMF/H_2O mixtures at the same concentration ($10^{-6}molL^{-1}$, excitation wavelength:327nm).

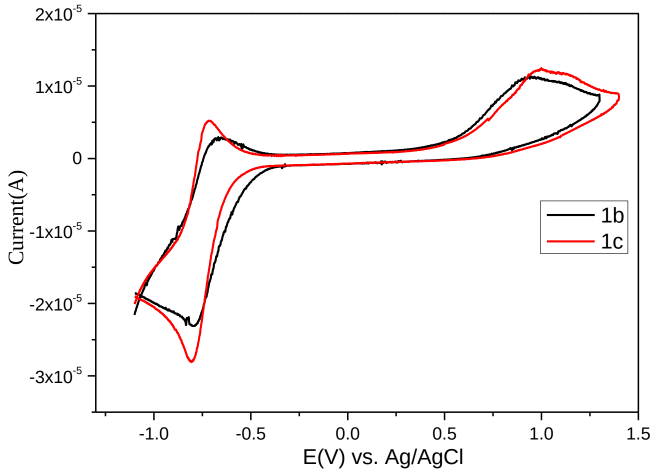

Figure S8 Cyclic voltammograms of **1a** in$CHCl_3$(1×10^{-3} M).

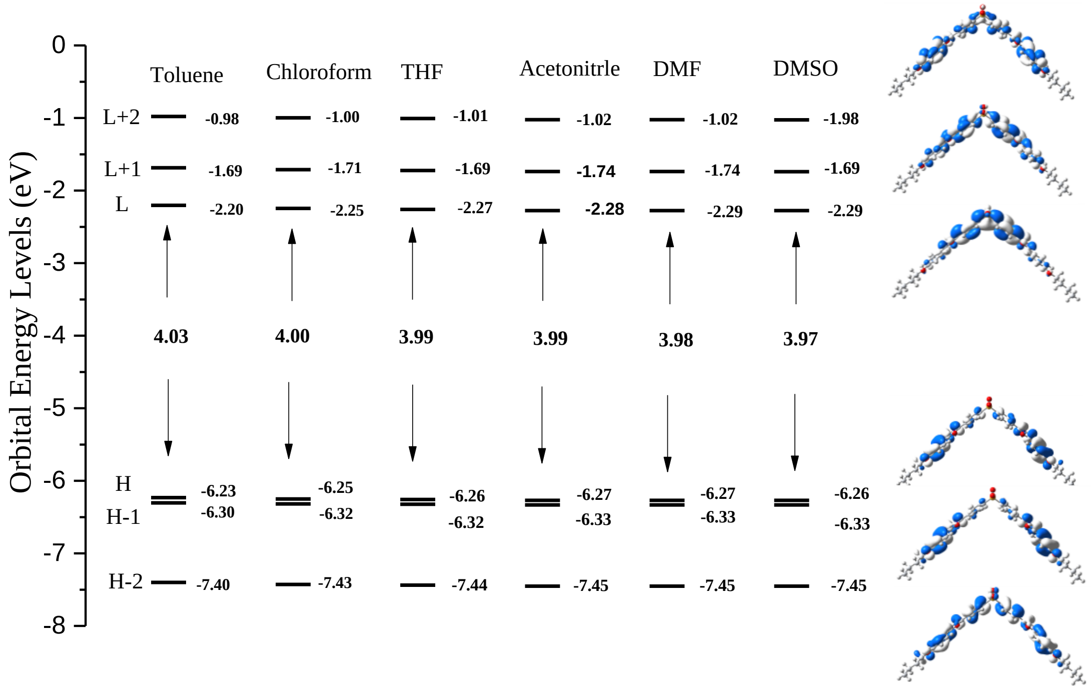

Figure S9 (1) Electron density contours and orbital energies calculated for the HOMOs and LUMOs of **1b** at the B3LYP/DZP level. H and L denote HOMO and LUMO, respectively.

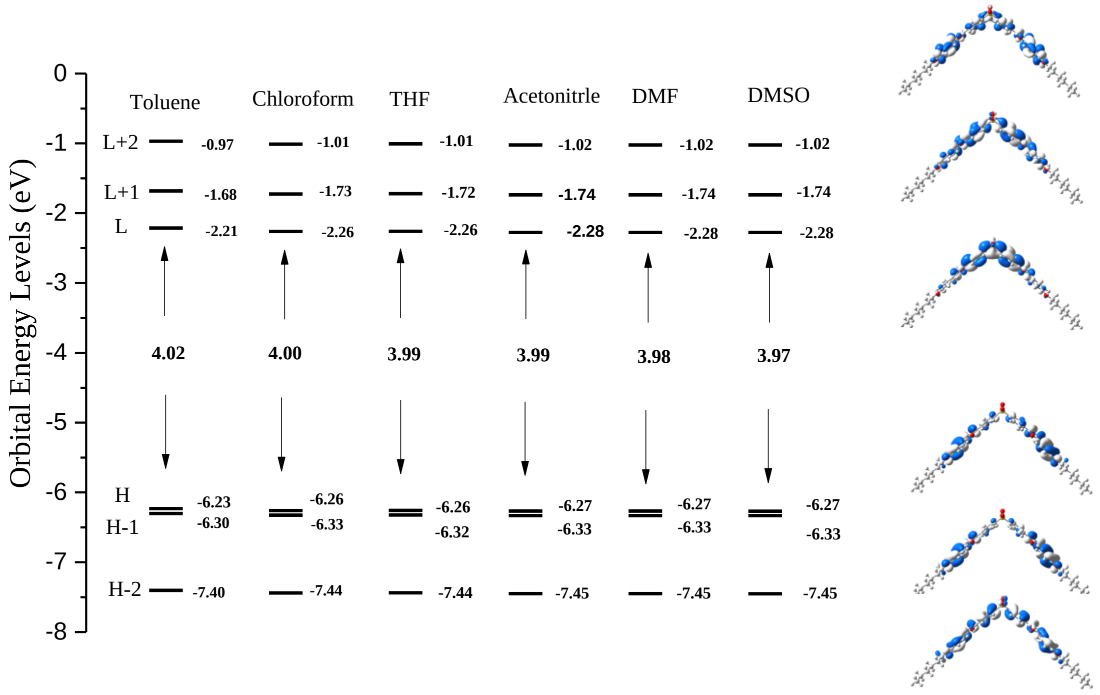

Figure S9 (2) Electron density contours and orbital energies calculated for the HOMOs and LUMOs of **1c** at the B3LYP/DZP level. H and L denote HOMO and LUMO, respectively.
